# Immunogenicity of biologic therapies for migraine: a review of current evidence

**DOI:** 10.1186/s10194-020-01211-5

**Published:** 2021-01-07

**Authors:** Joshua M. Cohen, Xiaoping Ning, Yoel Kessler, Michele Rasamoelisolo, Verena Ramirez Campos, Michael J. Seminerio, Lynda J. Krasenbaum, Honglue Shen, Jennifer Stratton

**Affiliations:** Teva Pharmaceuticals, West Chester, PA USA

**Keywords:** Migraine prevention, Prophylaxis, CGRP pathway–targeted monoclonal antibodies, Immunogenicity, Anti-drug antibodies

## Abstract

**Background:**

Monoclonal antibodies (mAbs) targeting the calcitonin gene-related peptide (CGRP) pathway have been shown to be effective in migraine prevention. Eptinezumab, erenumab, fremanezumab, and galcanezumb have shown efficacy in clinical trials along with favorable safety and tolerability profiles. Although erenumab is a human mAb and the others have been humanized to varying degrees, they all have the capacity to provoke immune reactions. The present review article aims to discuss the current relationship between mAbs targeting the CGRP pathway (CGRP mAbs) and immunogenicity and their potential clinical implications.

**Findings:**

The incidence of patients developing anti-drug antibodies (ADAs), their titer, and clinical significance are highly variable and depend on a variety of different drug and patient factors. Neutralizing ADAs (NAbs) bind to and inhibit or reduce the pharmacologic activity of the biologic drug molecule, whereas non-neutralizing antibodies (Non-NAbs) bind to the biologic drug molecule without affecting pharmacologic activity in an in vitro test, although pharmacokinetics and drug clearance may be affected. A direct comparison of immunogenicity data across clinical trials with different biologics is not possible due to a lack of standardized assays. Several phase 2, phase 3, and long-term studies evaluating CGRP mAbs for migraine prevention have reported immunogenicity data (5 studies each for eptinezumab, erenumab, fremanezumab, and galcanezumab). Across these studies, prevalence of ADAs varied, ranging from < 1% to ~ 18%. Neutralizing ADAs were slightly less common, with a prevalence ranging from 0 to 12%. Adverse events related to ADA formation were rare.

**Conclusions:**

As more CGRP mAb studies are conducted and more long-term follow-up data become available, evidence is increasing that immunogenicity rates of biologic therapies for migraine are low, and adverse events related to ADAs are rare. Taken together, these results add to the growing body of evidence for the safety and tolerability of this class of migraine medications.

## Background: biologic agents for migraine prevention

Migraine is a complex, debilitating neurologic disease that is a leading cause of disability worldwide, with an estimated global prevalence of 15% to 18% [1]. Prior to the development of calcitonin gene-related peptide (CGRP) pathway–targeted monoclonal antibodies (CGRP mAbs), medications available for migraine prevention were not specifically “targeted” to migraine and were generally underused, associated with poor tolerability, insufficient efficacy, and/or demonstrated very low rates of adherence and persistence [2]. CGRP plays an integral role in migraine pathogenesis, and studies have demonstrated that blockade of the CGRP ligand or its receptor can both treat and prevent migraine. Monoclonal antibodies (mAbs) targeting the CGRP pathway (ligand or receptor) represent a mechanism-specific targeted approach to migraine prevention and have proven to be safe, effective, and generally well tolerated [3, 4].

Currently 4 CGRP mAbs have been approved by the FDA for preventive treatment of migraine in adults: fremanezumab [5], erenumab [6], eptinezumab [7], and galcanezumab [8]. Treatment with a therapeutic protein, such as a mAb, can potentially lead to the induction of an immune response, resulting in the formation of anti-drug antibodies (ADAs) with or without neutralizing antibodies (NAbs) [9]. Depending on the type and the magnitude, immunogenic responses to biologic therapies may impact treatment efficacy and/or lead to adverse effects in patients [10, 11]. This review summarizes the various mechanisms of immunogenicity, the impact of ADAs/NAbs on mAb efficacy and safety, and the current clinical data on immunogenicity of CGRP mAbs and their clinical consequences.

### Mechanisms of immunogenicity

**Fig. 1:**
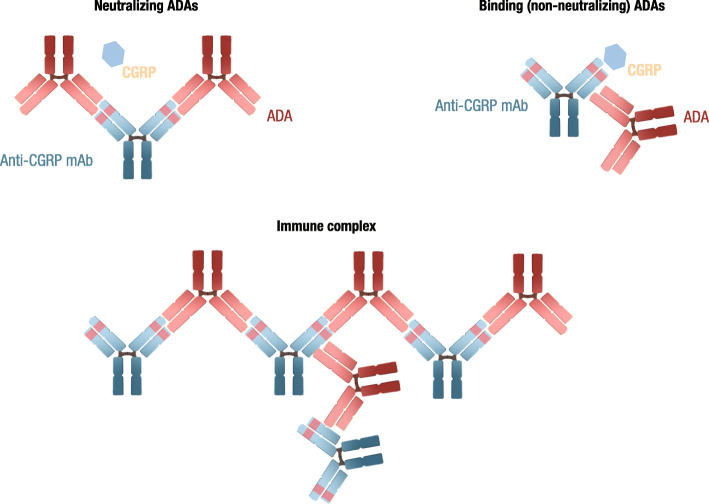
Types of immunogenic responses. Immunogenic responses can lead to the formation of ADAs, which are further classified as NAbs or non-NAbs. ADAs that interact with 1 or more additional ADA can form large structures known as immune complexes. The immune complex in this figure depicts both NAb and non-NAb ADAs. ADA, anti-drug antibody; CGRP, calcitonin gene-related peptide; mAb, monoclonal antibody; NAb, neutralizing antibody; non-NAb, non-neutralizing antibody

Biologic therapies, such as peptide-based therapies and mAbs, are increasingly used to treat a wide range of health conditions. Treatment with these biologics can lead to a variety of immune responses that range in severity [9]. ADAs are produced by B cells through both T cell–dependent and T cell–independent processes. T cell–independent ADA production generally involves a direct interaction between a therapeutic protein and cell surface B cell receptors, leading to internalization and B cell activation. It is also possible for specialized circulating dendritic cells to directly ingest a therapeutic protein, which is then presented to splenic B cells, resulting in ADA production. ADAs produced by T cell–independent processes are typically transient and have a lower affinity for the therapeutic protein; however, subsequent stimulation of B cells by additional antigens or epitopes may lead to longer lasting and/or higher titer ADA [12]. During T cell–dependent ADA production, antigen-presenting cells (APCs) derive peptides from a therapeutic protein, which are then presented to naïve CD4+ T cells, leading to T cell activation in a class II major histocompatibility complex (MHC)–restricted process [12]. Cytokines produced by activated T cells enhance B cell activation and expansion, resulting in longer lasting and higher affinity immunoglobulin G ADAs. Furthermore, B cell activation may also lead to the formation of plasma cells and memory B cells [13]. ADAs are broadly categorized as either NAb or non-neutralizing antibodies (non-NAbs) [14]. Non-NAbs bind to pharmacologically inactive sites on therapeutic proteins. Conversely, NAbs directly bind to therapeutic protein active sites, thereby potentially reducing efficacy (Fig. 1) [13, 15].

### Factors influencing immunogenicity and mitigation

Among biologic therapies, a wide range of immunogenicity rates have been reported, from < 1% to > 50% in some cases [11]. The immunogenicity of biologic therapies is influenced by a range of factors, including product-specific or treatment-related factors (eg, dose, route of administration, protein structure, protein stability, and formulation) and patient-related factors (eg, genetic predisposition, age, disease status, concomitant medication use, comorbidities) [10–12, 15]. Both the type and magnitude of ADAs produced can vary greatly between individuals, further affecting the extent to which ADAs impact each patient. Finally, ADA binding to a target therapeutic protein can lead to the formation of immune complexes that may significantly impact the extent to which ADAs and NAbs affect a given treatment [13]. It has been suggested that therapies administered by subcutaneous injection are more immunogenic than those administered intravenously, and this is thought to be related to the presence of cutaneous dendritic cells serving as APCs leading to T cell–dependent ADA production [11]. Therapeutic proteins containing human or fully humanized sequences tend to be less immunogenic than those containing murine or chimeric sequences; moreover, proteins with a high likelihood of forming immune complexes, containing epitopes recognized by class II MHC, and/or certain posttranslational modifications (eg, glycosylation) are more immunogenic than those lacking these elements [11, 12]. Due to a significant impact of peptide sequence on immunogenicity, a range of in silico and in vitro tools exist to identify and minimize risk when developing a biologic therapy. Biologic therapies can be evaluated to determine the risk of immunogenicity by identifying potential T cell epitopes using in silico analyses (eg, statistical, interference, or structural modeling) or in vitro T cell stimulation [16]. This approach provides an opportunity to engineer biologics, with the goal of increasing the humanness of the therapeutic protein.

### Potential consequences of immunogenicity on mAb safety and efficacy

NAbs are ADAs that directly interact with pharmacologically active regions of a mAb and thus may directly prevent or reduce mAb target binding, thereby potentially diminishing efficacy (Fig. 1). Although non-NAbs do not directly affect pharmacologic activity, both NAbs and non-NAbs can affect the systemic exposure of a therapeutic protein. For example, ADA/mAb interactions that increase clearance and shorten the elimination half-life may decrease therapeutic exposure [10, 13]. Conversely, non-NAb/mAb interactions that decrease clearance and lengthen the elimination half-life may increase therapeutic exposure [10, 13].

The extent of these effects on the efficacy of a therapeutic protein depends on many factors, including ADA kinetics (eg, onset and duration of immune response), titers, target affinity, and the propensity for immune complex formation [11, 13, 15, 17]. Immune complexes form when ADAs bind to therapeutic proteins, such as mAbs. Immune complexes are cleared through several mechanisms, with larger complexes being cleared more quickly than smaller complexes, which may persist longer [11, 13]. Depending on ADA affinity and titer, among other things, immune complexes can vary greatly in size and stability (Fig. 1) [13, 18]. For example, in cases where the titer and affinity of ADAs are low, immune complexes may be small or may form less readily and thus may have little or no impact on mAb efficacy or safety. Conversely, ADA reactions with high titers and/or high affinity for the therapeutic protein may favor the formation of large ADA therapeutic protein aggregates with more drastic impacts on systemic exposure or pharmacologic activity (eg, interaction between therapeutic mAb and target protein/receptor) [18, 19]. Altogether, these mechanisms may result in loss of efficacy, altered pharmacokinetics, cross-reactivity to endogenous protein, and hypersensitivity reactions, such as infusion reactions to anaphylactic reactions [13, 20, 21].

Immunologic responses to therapeutic proteins can cause adverse reactions, which have a range of different phenotypes (clinical presentations) and endotypes (underlying cellular and molecular mechanisms of response). Hypersensitivity reactions manifest with a range of mild to severe symptoms depending on the underlying mechanisms of immune response (described as types I–IV, according to the revised Gell and Coombs’ classification) and may present at any time from treatment initiation through several months following the abrogation of treatment [11]. Type I reactions are commonly mediated by immunoglobulin E antibodies, although they can also occur independent of immunoglobulin E release through T cells. These reactions usually occur within minutes to hours after treatment and can typically be prevented by prophylactic antihistamine treatment. Symptoms of type I hypersensitivity reactions can include pruritus, flushing, shortness of breath, rash, and hypertension; in severe cases, type I reactions can involve life-threatening anaphylaxis [13, 22, 23]. Therapeutic proteins can bind to cell surface protein targets and attract circulating ADAs, leading to formation of immune complexes on cell membranes in tissues. Type II reactions result from the adverse effects of these ADA/immune complexes on cell membranes [13]. Type III reactions occur when therapeutic proteins bind soluble antigens and aggregate to form immune complexes that are not cleared from the body [23]. These immune complexes may precipitate and deposit, particularly in diffuse capillary networks or tissues high in fenestrated epithelium (eg, kidneys and synovial membrane) resulting in complement activation, inflammation, and local (eg, nephropathy/nephritis) or systemic injury [13, 22, 23]. Type IV reactions, also called delayed type IV hypersensitivity, are thought to be mediated by T cell activation (though other mechanisms may be involved), typically arise 12 h to several weeks following mAb treatment, and may be mild (rash) or severe (Stevens-Johnson syndrome and toxic epidermal necrolysis) [13, 23]. It is noteworthy that type I and type III hypersensitivity reactions are most common with respect to ADAs [22]. With respect to fremanezumab, 2 mutations were introduced into the constant region of the heavy chain to limit normal antibody effector functions. This loss of function mutation was designed to prevent stimulation of antibody-dependent cell-mediated cytotoxicity and triggering of complement-dependent cytotoxicity [24].

### CGRP pathway–targeting mAbs and immunogenicity: immunogenicity incidence

There are many challenges to evaluating ADAs and understanding the impact of ADA on the efficacy and safety of biologic therapies. For example, several ADA assay formats are available and, as such, a consensus method for detecting, measuring, and reporting ADAs has not been established [15, 25]. In addition, the reagents required to test for ADAs are specific to each therapeutic protein. This lack of a uniform detection method makes it challenging to interpret similarities and differences in immunogenicity among different biologics, despite similarities in target and/or mechanism of action [10]. Biologic therapies that bind soluble ligands make up a large proportion of antibody therapeutics. Drug targets, when present at sufficiently high circulating concentrations, can potentially interfere with the performance of ADA assays [26]. In this context, eptinezumab, fremanezumab, and galcanezumab bind to the soluble ligand, CGRP, whereas erenumab is a mAb that binds to the CGRP receptor [27]. Nevertheless, in order to provide an overview of the current evidence on ADAs and NAbs in migraine preventive biologics, we have briefly summarized published results from recent migraine prevention clinical trials evaluating mAbs that target the CGRP pathway.

#### Eptinezumab

**Fig. 2:**
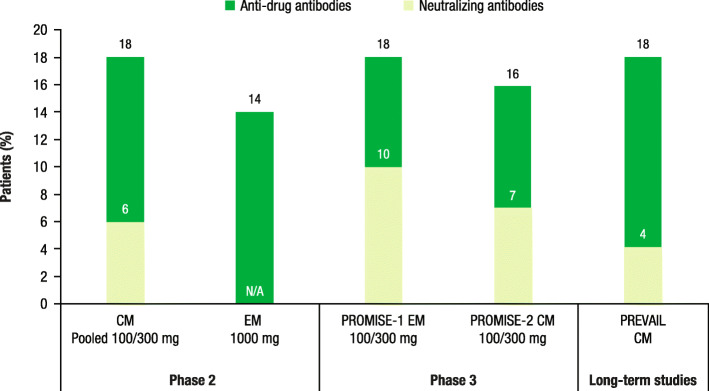
Anti-drug antibodies and neutralizing antibodies in eptinezumab trials [28–32]. CM, chronic migraine; N/A, data not available; EM, episodic migraine

In a phase 2 trial of patients with chronic migraine (CM) treated with eptinezumab (either 100 or 300 mg), 18% of patients developed ADAs and 6% of patients developed NAbs (Fig. 2) [28]. In a separate phase 2 study of patients with episodic migraine (EM) treated with eptinezumab 1000 mg, 14% of patients developed ADAs [29]. In the two phase 3 PROMISE trials of eptinezumab (100 or 300 mg), among patients with EM or CM, 18% and 16% of patients developed ADAs, respectively, whereas 10% and 7% of patients developed NAbs, respectively [30, 31]. In an open-label PREVAIL study, among patients with CM, at Week 24, 18% of patients receiving eptinezumab (300 mg) had ADAs and 4% had NAbs (Fig. 2) [32].

#### Erenumab

**Fig. 3:**
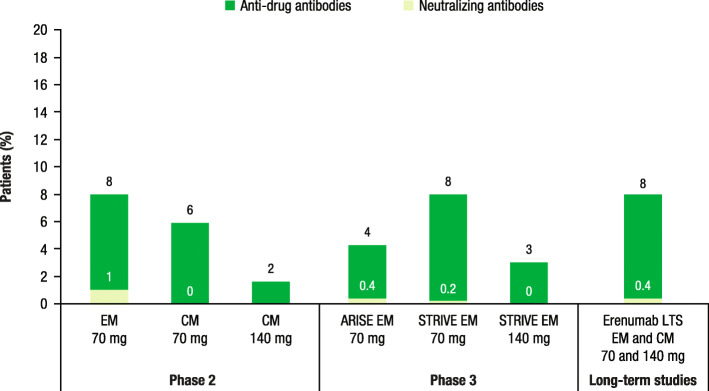
Anti-drug antibodies and neutralizing antibodies in erenumab trials [33–38]. EM, episodic migraine; CM, chronic migraine; LTS, long-term study

Among patients with EM receiving erenumab (70 mg) during a phase 2 study, 8% developed ADAs and 1% developed NAbs (Fig. 3) [33]. In a separate phase 2 study of patients with CM, 6% and 2% of patients developed ADAs with erenumab 70 and 140 mg, respectively; none of the patients developed anti-erenumab NAbs [34]. In the phase 3 ARISE trial of patients with EM receiving erenumab (70 mg), 4% developed ADAs and 0.4% developed NAbs [35]. Furthermore, in the phase 3 STRIVE trial, among patients with EM receiving erenumab 70 mg, 8% developed ADAs and 0.2% developed NAbs compared with 3% and 0% of patients receiving erenumab 140 mg [36]. In the long-term extension studies of 4 placebo-controlled trials, among patients with EM or CM receiving 70 or 140 mg of erenumab, 8% developed ADAs and 0.4% developed NAbs (Fig. 3) [37, 38].

#### Fremanezumab

**Fig. 4:**
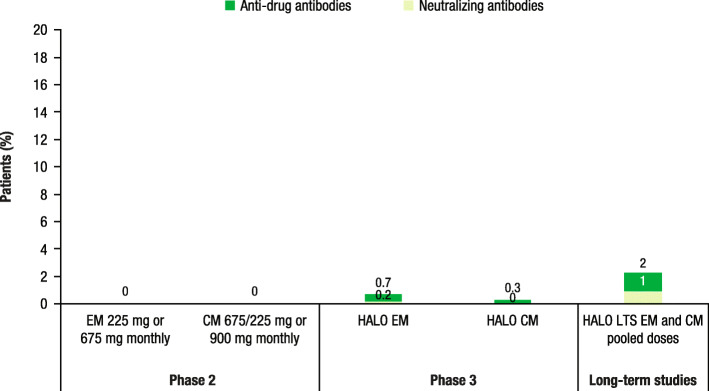
Anti-drug antibodies and neutralizing antibodies in fremanezumab trials [2, 39–42]. EM, episodic migraine; CM, chronic migraine; LTS, long-term study

Among patients with EM or CM who received fremanezumab quarterly or monthly during phase 2 trials, no patients developed ADAs or NAbs (Fig. 4) [39, 40]. Among those receiving fremanezumab quarterly or monthly in phase 3 studies, 0.7% of patients in the HALO EM trial and less than 0.3% of patients in the HALO CM trial developed ADAs; 0.2% of patients developed NAbs in the HALO EM study and no patients developed NAbs in the HALO CM trial [2, 41]. Finally, among patients in the open-label HALO LTS study, 2% of patients developed ADAs and 1% of patients developed NAbs (Fig. 4) [42].

#### Galcanezumab

**Fig. 5:**
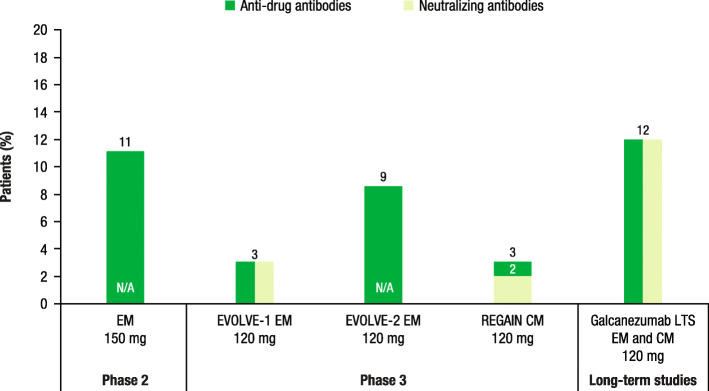
Anti-drug antibodies and neutralizing antibodies in galcanezumab trials [43–47]. EM, episodic migraine; N/A, data not available; CM, chronic migraine; LTS, long-term study

In a phase 2 study of patients with EM receiving galcanezumab 150 mg, 11% of patients developed ADAs; however, the data on number of patients who developed NAbs was not reported (Fig. 5) [43]. Results from the EVOLVE-1 phase 3 study of patients with EM receiving galcanezumab 120 mg showed that 3% of patients developed ADAs and 3% developed NAbs [44]. In the EVOLVE-2 phase 3 study, 9% of patients receiving galcanezumab 120 mg developed ADAs while 5% and 1% of patients developed ADAs in the galcanezumab 240 mg and placebo groups, respectively [45]. Of the 32 patients who developed ADAs in the 3 treatment groups, 29 of them had neutralizing ADAs present. Furthermore, in the phase 3 study, REGAIN, among patients with CM receiving galcanezumab 120 mg, 3% of patients developed ADAs and 2% developed NAbs [46]. Finally, results from a long-term, open-label study of patients with EM or CM receiving galcanezumab 120 mg showed that 12% of patients developed ADAs, all of whom also developed NAbs (Fig. 5) [47].

### Adverse events among patients with ADAs in HALO trials

In the HALO EM trial, of the 4 patients receiving fremanezumab who developed ADAs, 2 patients had no adverse events (AEs): 1 patient had 2 transient events of injection-site pain and also experienced 1 event of a mild decrease in hemoglobin with no evidence of hemolysis, and 1 patient who developed ADAs and NAbs experienced an upper respiratory infection. During the HALO CM trial, 2 patients developed ADAs following treatment with fremanezumab: 1 patient had no AEs during the study and the other patient experienced 2 transient events of injection-site pain, 1 event of mild injection-site induration, and 1 event of mild injection-site erythema. Finally, among 43 patients who developed ADAs in the HALO LTS study, including 18 with NAbs, there were no apparent safety consequences nor any impact on efficacy. None of the patients in the HALO studies who developed ADAs had a severe hypersensitivity reaction.

Although patient-level data is not available for other CGRP mAbs described in this review, publications and prescribing information suggest that ADAs formed did not have a significant impact on efficacy or safety [6–8]. Taken together, no AEs potentially related to ADAs have been identified with this class of drugs.

## Discussion

mAbs have led to a paradigm shift in the management of migraine. Evidence from clinical trials demonstrates the efficacy and safety of mAbs targeting the CGRP pathway. As with all mAb therapies, there is a risk for adverse reactions, including the development of NAbs that may diminish treatment efficacy. As described above, several ADA assay formats are available and, as such, a consensus method for detecting, measuring, and reporting ADAs has not been established [15, 25]. The lack of a uniform ADA detection method and the reagents that are specific to each biologic therapy make it challenging to interpret similarities and differences in immunogenicity among different biologics, despite similarities in target and/or mechanism of action [10]. Current evidence from the literature shows that there is no apparent effect of ADAs on safety and efficacy of CGRP mAbs. Nevertheless, for all CGRP mAbs, the prescribing information contains warnings about hypersensitivity reactions [5–8]. With regards to erenumab, there are also warnings about constipation and hypertension [6]. Furthermore, the European labels for galcanezumab and erenumab also include warnings about anaphylaxis [48, 49].

Among clinical studies of eptinezumab, erenumab, fremanezumab, and galcanezumab, reported rates of ADAs and NAbs were lowest in fremanezumab studies, whereas studies with eptinezumab had the highest reported rates of ADAs and NAbs. This observation is noteworthy given that eptinezumab is administered intravenously, whereas erenumab, fremanezumab, and galcanezumab are administered by subcutaneous injection. Currently available data on immunogenicity profiles do not demonstrate an impact of ADA development on the efficacy or safety of CGRP mAbs. This could be attributed to the low ADA titer of the already low ADA incidence. Nevertheless, ongoing trials and upcoming studies can provide additional information about immunogenicity; indeed, there are questions about the impact of NAbs and ADAs on mAb efficacy over the long-term, especially whether loss of drug efficacy is associated with an increase in ADA titer over time. There are also questions about long-term safety concerns in patients developing ADAs, particularly with respect to instances where immune complexes form and whether they lead to cytokine activation, precipitate out and deposit in tissues, and lead to additional tissue injury (nephropathy/nephritis).

## Conclusions

Results from clinical trials among patients receiving CGRP mAbs for migraine prevention have demonstrated that treatments are generally well tolerated and effective. The prevalence of ADAs among CGRP mAb clinical trials range from < 1% to ~ 18%, depending on patient population, biologic therapy, and treatment paradigm. Among the same studies, NAbs were slightly less common, with a prevalence ranging from 0 to 12%. While immunogenicity rates were low, available evidence shows that immunogenicity-related AEs were rare, thus underscoring the safety of CGRP mAbs. Additional data could help to understand the long-term impacts of immunogenicity on CGRP mAb efficacy and safety.

## Data Availability

Not applicable.

## Abbreviations

ADA: Anti-drug antibody
AE: Adverse event
APC: Antigen-presenting cell
CGRP: Calcitonin gene-related peptide
CGRP mAbs: Calcitonin gene-related peptide pathway–targeted monoclonal antibodies
CM: Chronic migraine
EM: Episodic migraine
FDA: Food and Drug Administration
LTS: Long-term study
mAb: Monoclonal antibody
MHC: Major histocompatibility complex
NAb: Neutralizing antibody
Non-NAb: Non-neutralizing antibody
